# Supplementary material for: Elucidating the origins of multimode vibrational coherences of polyatomic molecules induced by intense laser fields
Source: Nat Commun. 2017 Sep 29;8:735. doi: 10.1038/s41467-017-00848-2 (PMC5622070; doi:10.1038/s41467-017-00848-2)
Supplement: Supplementary file 1 — Supplementary Information [file 41467_2017_848_MOESM1_ESM.pdf]

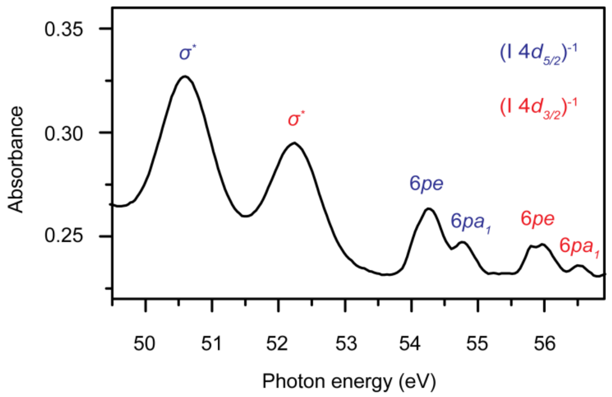

Supplementary Figure 1. I 4*d* transitions of CH_3I. Static I 4*d* core-level photoabsorption spectrum of CH_3I measured with the experimental apparatus.

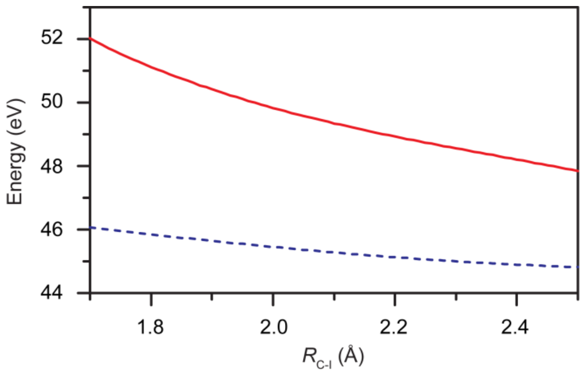

Supplementary Figure 2. I 4*d* inner-shell transition energies with C—I bond distance. Calculated I 4*d* inner-shell transition energies for CH_3I (red line) and CH_3I^+ (blue dashed line) as a function of C—I bond distance.

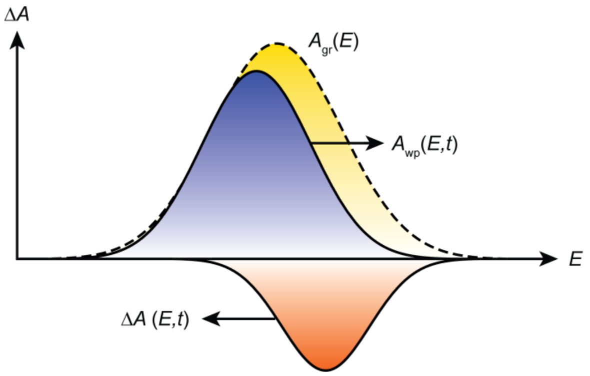

Supplementary Figure 3. The phase of vibrational wave packet. Schematic illustration of the ground-state static absorption spectrum $A_{\mathrm{gr}}(E)$, the instantaneous absorption spectrum of the vibrational wave packet $A_{\mathrm{wp}}(E,t)$, and the differential absorption spectrum $\Delta A(E,t)$.

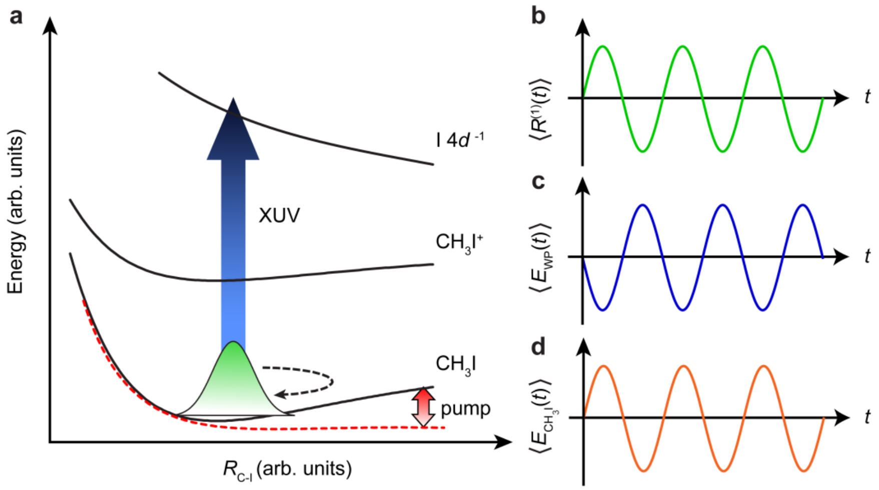

Supplementary Figure 4. C—I stretching vibrational wave packet induced by bond softening. (a) An intense laser field induces softening of the neutral CH_3I potential along the C—I stretch coordinate. (b) The equilibrium vibrational probability density initially moves towards larger $R_{\mathrm{C-I}}$ values. (c) The XUV transition energy of the wave packet $\langle E_{\mathrm{wp}}(t)\rangle$ initially decreases. (d) The spectral first moment $\langle E_{\mathrm{CH_3I}}(t)\rangle$ exhibits an initial increase, resulting in a phase of $\phi_{\mathrm{CH_3I}} = -\pi/2$ rad.

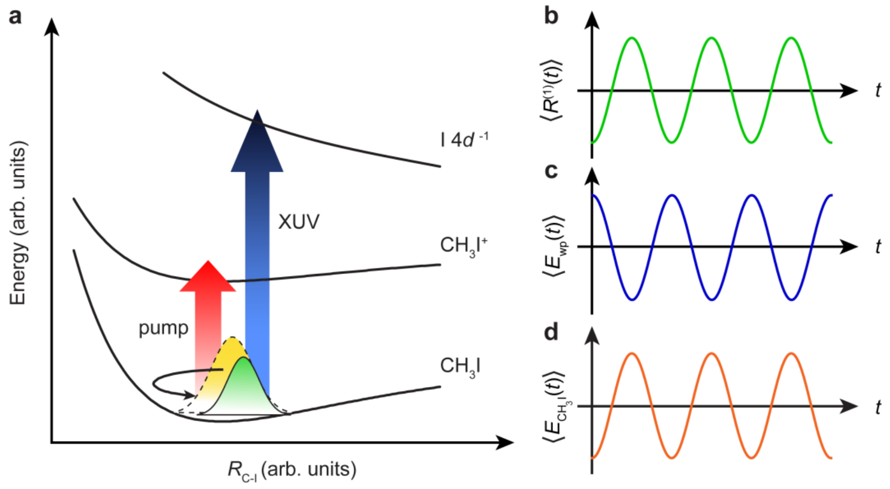

Supplementary Figure 5. C—I stretching vibrational wave packet induced by *R*-selective depletion. (a) An intense laser field selectively depletes the subset of molecules with shorter C—I bond distances via *R*-selective depletion. (b) The residual vibrational probability density near the outer turning point initially moves towards smaller R_{CI} values. (c) The XUV transition energy of the wave packet $\langle E_{\mathrm{wp}}(t)\rangle$ initially increases. (d) The spectral first moment $\langle E_{\mathrm{CH_3I}}(t)\rangle$ exhibits an initial decrease, resulting in a phase of $\phi_{\mathrm{CH_3I}} = 0$ rad.

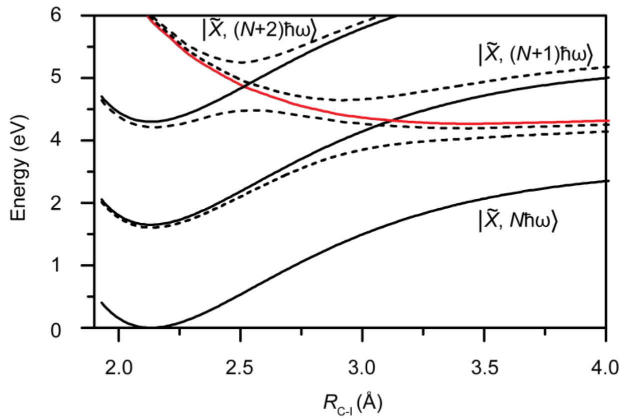

Supplementary Figure 6. Floquet picture of bond softening. Manifold of Floquet states $|\tilde{X}, n\hbar\omega\rangle$ (black lines; $n = N$ to $N + 2$) crossing the $^3Q_{0^+}(A_1)$ excited state (red line). The resultant adiabatic curves (dashed lines) reveal the creation of avoided crossings and the distortion of the potential energy curves by intense laser fields.

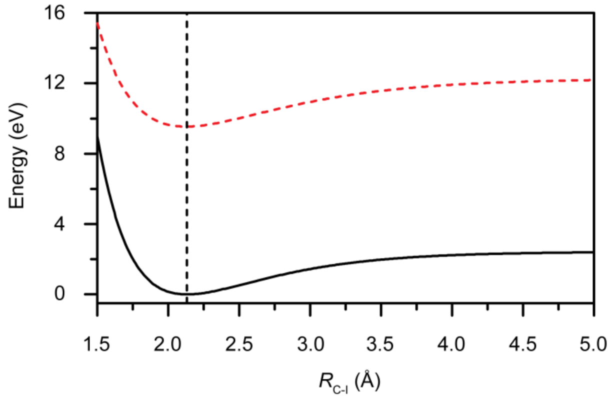

Supplementary Figure 7. Morse potentials for CH_3I and CH_3I^+. Morse potentials for the electronic ground states of CH_3I (solid black line) and CH_3I^+ (red dashed line). The parameters used to construct these potentials are given in Supplementary Table S2. The vertical dashed lines denote the equilibrium C—I bond distance of CH_3I.

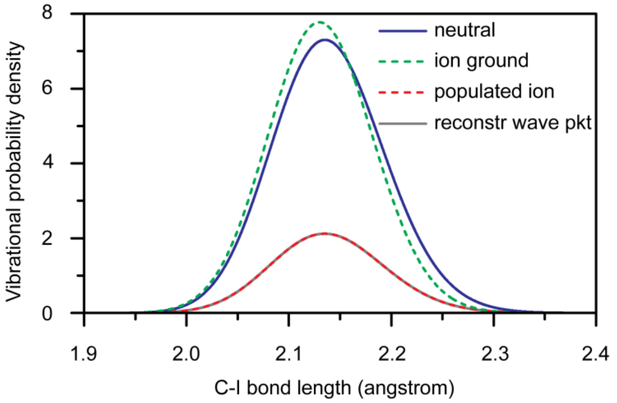

Supplementary Figure 8. The C—I stretching vibrational wave packet from displacive excitation. Vibrational probability density plots along the C—I stretch coordinate for the initial CH_3I molecule at thermal equilibrium (solid blue line), the CH_3I^+ fraction produced by ionization (red dashed line), and the CH_3I^+ $v = 0$ level (green dotted line). The initial vibrational probability density reconstructed for the wave packet (grey line) agrees well with that of CH_3I^+ produced by ionization.

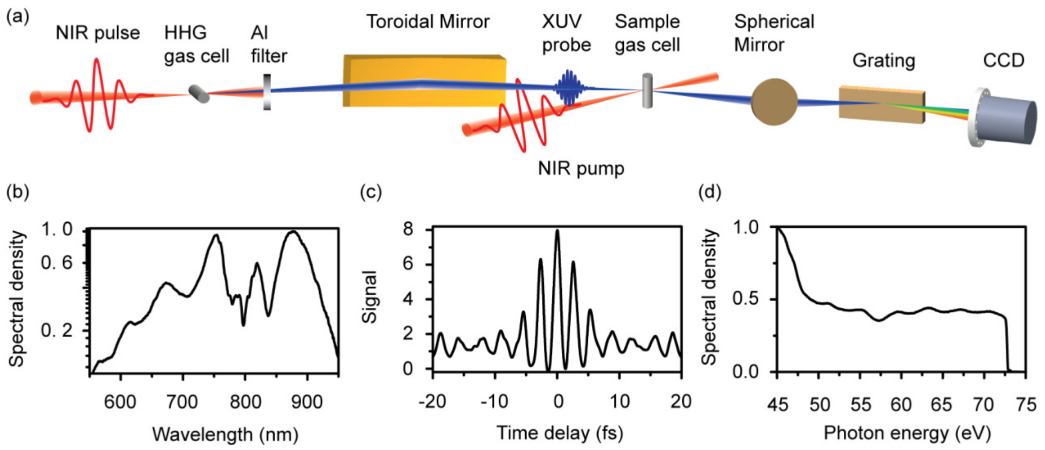

Supplementary Figure 9. Femtosecond XUV transient absorption setup. (a) Schematic illustration of the experimental setup for femtosecond time-resolved XUV transient absorption spectroscopy. (b) The corresponding laser spectrum on a logarithmic scale. (c) Measured interferometric autocorrelation trace of a 5.6-fs FWHM laser pulse. (d) XUV spectrum generated from argon gas via HHG. The spectrum beyond 72.64 eV is blocked by the L_3 edge cut-off of the Al foils employed in the experiments.

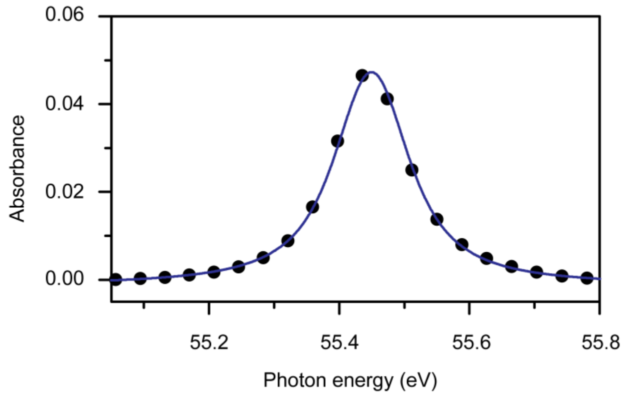

Supplementary Figure 10. Spectral resolution of XUV spectrometer. Fit of the Xe^+ ${}^2P_{3/2} \rightarrow {}^2D_{5/2}$ transition at 55.4 eV to a Voigt profile, revealing a spectrometer resolution of 47 meV FWHM.

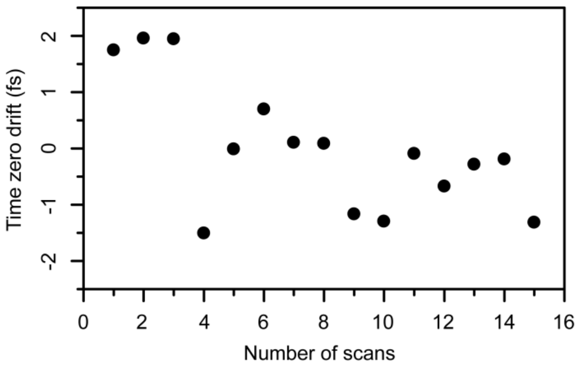

Supplementary Figure 11. Timing jitter of the XUV transient absorption setup. Determination of the jitter in time-zero, as determined by the rise of the CH_3I^+ ($\tilde{X}^+\ ^2E_{3/2}$) parent ion signal at 47.0 eV. The standard deviation gives a timing jitter of $\Delta t_0 = 1.2$ fs.

Supplementary Table 1. I 4*d* transitions of CH_3I. Observed I 4*d* core-level transitions and transition energies. Values in parentheses are obtained from ref. 1.

Transition	Transition energy (eV)
I $4d_{5/2} \rightarrow \sigma^*_{\mathrm{C-I}}$	50.62 (50.62)
I $4d_{3/2} \rightarrow \sigma^*_{\mathrm{C-I}}$	52.25 (52.34)
I $4d_{5/2} \rightarrow 6pe$	54.25 (54.29)
I $4d_{5/2} \rightarrow 6pa_1$	54.80 (54.84)
I $4d_{3/2} \rightarrow 6pe$	55.94 (56.05)
I $4d_{3/2} \rightarrow 6pa_1$	56.53(56.57)

Supplementary Table 2. Parameters for CH_3I and CH_3I^+ Morse potentials. Summary of the spectroscopic parameters used to reconstruct the Morse potentials

	CH_3I $\tilde{X}$ $({}^1A_1)$	CH_3I^+ $\tilde{X}^+$$({}^2E_{3/2})$
I_p / eV	9.5381 (ref. 9)	–
D_0 / eV	2.431 (ref. 9)	2.731 (ref. 9)
ω_0 / cm^{-1}	533 (ref. 10)	480 (ref. 11)
r_e / Å	2.132 (ref. 12)	2.126 (ref. 13)

Supplementary Note 1: Static I 4*d* photoabsorption spectrum of CH_3I

The static photoabsorption spectrum of CH_3I collected about the I 4*d* absorption edge is shown in Supplementary Figure 1. Two sets of peaks are visible; they arise from transitions between the spin-orbit-split I 4*d* core levels – $4d_{5/2}^{-1}$ and $4d_{3/2}^{-1}$ – to either the C—I σ^* level or the iodine-centered 6*p* Rydberg states. The observed transitions and the measured transition energies, along with those obtained from inner-shell electron energy loss spectroscopy[1], are summarized in Supplementary Table 1.

Supplementary Note 2: Ab initio simulations of iodine 4*d* inner-shell transition energies

Mapping of the measured first-moment modulation in the transient absorption signal of CH_3I $\langle E_{\mathrm{CH_3I}}(t)\rangle$ to the time-dependent motion of the wave packet $\langle R^{(1)}(t)\rangle$ along the C—I stretching coordinate requires knowledge of how the XUV probe transition energy varies with the C—I bond distance. To this end, we employ time-dependent density functional theory (TD-DFT) within the GAUSSIAN software package[2] to calculate the iodine 4*d* core level transition energies as a function of C—I bond distance. The potential energy scan along the C—I bond stretching coordinate and the TDDFT calculations both employ the wB97X exchange-correlation functional and the Def2TZVP basis set[3,4]. The resultant I 4*d* → σ^*(C—I) and I 4*d* → 5*pe* transition energies calculated as a function of C—I bond distance for CH_3I and CH_3I^+, respectively, are shown in Supplementary Figure 2.

Supplementary Note 3: Anti-correlation between transition energy shift and first-moment time trace

The first-moment time trace $\langle E_{\mathrm{CH_3I}}(t)\rangle$ is computed about the ground-state bleaching region, where the differential absorption signal $\Delta A(E,t)$ is negative due to the depletion of the neutral species (E is the probe photon energy and t is the time delay). Because $\Delta A(E,t) = A_{\mathrm{wp}}(E,t) - A_{\mathrm{gr}}(E)$, where $A_{\mathrm{wp}}(E,t)$ is the time-dependent absorption spectrum of the

vibrational wave packet launched in the residual neutral species and $A_{\mathrm{gr}}(E)$ is the time-independent (static) absorption spectrum of the CH_3I (see Supplementary Figure 3), the directions of spectral shifts of $A_{\mathrm{wp}}(E,t)$ and $\Delta A(E,t)$ are anti-correlated. That is, a blue-shift of $A_{\mathrm{wp}}(E,t)$ leads to a red-shift of $\Delta A(E,t)$ and vice versa.

Supplementary Note 4: First-moment oscillation phases due to bond softening and *R*-selective depletion

Here, we consider how bond softening and R-selective depletion manifest themselves in the initial phase of the C—I stretching vibrational wave packet signal for neutral CH_3I. In the quasi-static picture of bond softening, irradiation of the molecule by an intense laser pulse transiently distorts the potential energy surface of the molecule[5]. As a result, the equilibrium vibrational probability density is displaced towards larger C—I distances, hence initiating wave packet motion (Supplementary Figure 4a). The time-evolution of the expectation value of the C—I distance, $\langle R^{(1)}(t)\rangle$, is shown in Supplementary Figure 4b. Given the monotonic decrease in the XUV transition energy with C—I distance, we expect the XUV transition energy of the wave packet, $\langle E_{\mathrm{wp}}(t)\rangle$, to show an initial decrease with time delay (Supplementary Figure 4c). Finally, because of the anti-correlation between the shift of the wave packet absorption energy and $\langle E_{\mathrm{CH_3I}}(t)\rangle$, we expect $\langle E_{\mathrm{CH_3I}}(t)\rangle$ to exhibit an initial phase of $\phi_{\mathrm{CH_3I}} = -\pi/2$ rad (Supplementary Figure 4d).

In R-selective depletion, we first assume that strong-field ionization selectively depletes the population near the inner turning point of the C—I stretching potential (Supplementary Figure 5a); this assumption is consistent with the steep decrease in the ionization potential for C—I bond lengths shorter than the equilibrium value (see inset of Figure 3 in the main text). As a result, the vibrational wave packet is launched from the outer turning point. The initial decrease of $\langle R^{(1)}(t)\rangle$ with wave packet motion (Supplementary Figure 5b) leads to an

increase of $\langle E_{\mathrm{wp}}(t)\rangle$ (Supplementary Figure 5c), and therefore, a decrease of $\langle E_{\mathrm{CH_3I}}(t)\rangle$, i.e., the initial phase should be $\phi_{\mathrm{CH_3I}} = 0$ rad (Supplementary Figure 5d).

Supplementary Note 5: Floquet picture of bond softening

In the main text, we consider how the quasi-static picture of bond softening leads to vibrational wave packet motion along the C—I stretching coordinate. The quasi-static approximation is valid for the high-intensity, low-frequency laser fields used in the present experiments. Here, we show that the complementary Floquet picture of bond softening[6] can also explain the launching of vibrational wave packets along the C—I stretching coordinate. Supplementary Figure 6 shows the manifold of field-dressed $\tilde{X}$ states, $|\tilde{X}, n\hbar\omega\rangle$ ($n = N$ to $N + 2$) (black lines), crossing the ${}^3Q_{0^+}(A_1)$ (red line) excited state. The ${}^3Q_{0^+}$ excited state dominates the A band transition[7] of CH_3I. The $\tilde{X}$ state potential corresponds to the Morse potential reconstructed from spectroscopic parameters (see Supplementary Note 6 below), whereas the ${}^3Q_{0^+}$ state potential is retrieved from ab initio simulations reported in ref. 8. It is evident from the adiabatic curves (dashed lines) that intense laser fields can introduce avoided crossings and distort the potential energy curve of the CH_3I $\tilde{X}$ state along the C—I stretching coordinate, hence launching vibrational wave packet dynamics.

Based on symmetry requirements, the one-photon crossing between the ground state and the ${}^3Q_{0^+}$ state requires the laser field to be aligned parallel to the molecular axis. In the case of the two-photon crossing, coupling between the ground and ${}^3Q_{0^+}$ states occurs when the laser field is aligned either parallel or perpendicular to the molecular axis.

Supplementary Note 6: Morse potential parameters for the electronic ground states of CH_3I and CH_3I^+

The spectroscopic parameters that were employed to reconstruct the Morse potentials for the CH_3I $\tilde{X}\,({}^1A_1)$ state and the CH_3I^+ $\tilde{X}^+({}^2E_{3/2})$ state are summarized in Supplementary Table

2 below. The reconstructed Morse potentials are shown in Supplementary Figure 7 with solid black line and red dashed line, respectively.

Note that reconstruction of Morse potentials requires the dissociation energy D_e, which in turn requires the zero-point energies of the reactant and product, in addition to D_0. In the absence of accurate values for the zero-point energies, we instead use D_0 to generate the Morse potentials. Since these potentials are used in R-selective depletion calculations, which uses as input the R-dependent energy gap between CH_3I and CH_3I^+, the error that results from the omission of the zero-point energies is expected to be small.

Supplementary Note 7: *R*-selective depletion simulations

We perform R-selective depletion calculations to verify that the difference in equilibrium C—I bond lengths of 0.006 Å can still support displacive excitation of the wave packet along the v_3 mode, even with thermal averaging and the finite focal volume. First, the various vibrational levels of the v_3 mode for neutral CH_3I are populated according to the Boltzmann distribution at a sample temperature of 353 K. The resultant normalized vibrational probability density, constructed from an incoherent sum of the probability densities of Morse oscillator wave eigenfunctions for $v = 0$ to $v = 3$, is plotted as the solid blue line in Supplementary Figure 8. That is,

$$|\Psi_{CH_3I}(R,0)|^2 = \sum_{v=0}^{3} c_v^2 |\psi_v(R)|^2 , \tag{1}$$

where R is the C—I coordinate, c_v^2 is the Boltzmann weight for the vibrational level v, and $\psi_v(R)$ is the Morse oscillator eigenfunction for the vibrational level v. Accounting for the anharmonicity of the C—I stretching potential (x_{33} = –3.44 cm^{-1})[14], the Boltzmann weights are found to be 0.8860, 0.1006, 0.0117, and 0.0014 for the first four vibrational levels.

Second, the R-dependent ionization rates are applied to deplete the vibrational probability density of the neutral. The Ammosov-Delone-Krainov[15] ionization rates employed here are

likely to be a good approximation, given that ionization involves the lone-pair electrons of iodine with predominantly atomic $5p$ character. Note that the ionization fraction is obtained by integrating the ionization rate $W_{\text{ion}}(R,t,r)$ over the temporal profile of the strong-field-ionizing pump pulse (t) and accounts for the transverse spatial profile (r) of the strong-field-ionizing pump and XUV probe beams, i.e., the calculated R-dependent ionization fraction, $f_{\text{ion}}(R)$, accounts for the varying spatial and temporal profiles of the pump beam, as well as the varying spatial profile of the probe beam:

$$f_{\text{ion}}(R) = \frac{\iint dt\, dr\ W_{\text{ion}}(R,t,r) \times 2\pi r\, A_{\text{XUV}}(r)}{\int dr\, 2\pi r\ A_{\text{XUV}}(r)} \times \left|\Psi_{\text{CH}_3\text{I}}(R,0)\right|^2, \quad (2)$$

where $A_{\text{pr}}(r)$ is the transverse profile of the XUV probe beam. The plot of $f_{\text{ion}}(R)$ is shown in Supplementary Figure 8 as the dashed red line. The peak of $f_{\text{ion}}(R)$ at 2.135 Å is displaced from the peak of the vibrational probability density for the $v = 0$ state of the CH_3I^+ ion (dotted green line in Supplementary Figure 8) at 2.130 Å by 0.005 Å. This finite displacement, though small, supports the observed displacive excitation of the C—I stretching wave packet.

The initial vibrational probability density created in the ion state, $f_{\text{ion}}(R)$, being a nonstationary state, can be described as a coherent superposition of vibrational eigenstates of CH_3I^+ along the C—I stretching coordinate, i.e.,

$$f_{\text{ion}}(R) = \left|\sum_{v} c_{\text{ion},v}\, \psi_{\text{ion},v}(R)\right|^2, \quad (3)$$

where $c_{\text{ion},v}$ is the amplitude coefficient and $\psi_{\text{ion},v}(R)$ is the Morse oscillator eigenfunction for the ion C—I stretching mode in the vibrational level v. Our analysis shows that the $f_{\text{ion}}(R)$ obtained from R-selective depletion simulations (see above) can be reasonably represented by a coherent superposition comprising the four lowest eigenstates, with coefficients $c_{\text{ion},0} = 0.99646$, $c_{\text{ion},1} = 0.07000$, $c_{\text{ion},2} = 0.04483$, and $c_{\text{ion},3} = 0.01234$ (see solid grey line in Supplementary Figure 8). The resultant wave packet,

$$\Psi_{\text{ion}}(R,t) = \sum_{v=0}^{3} c_{\text{ion},v}\, \psi_{\text{ion},v}(R)\, e^{-iE_{\text{ion},v}t/\hbar}\,, \tag{4}$$

where $E_{\text{ion},v}$ is the eigenenergy of the vibrational level v, can be used to compute the time-evolution of the C—I bond distance, $\langle R^{(1)}(t)\rangle$. Consistent with the displacive mechanism and the relative equilibrium C—I bond distances of CH_3I and CH_3I^+, $\langle R^{(1)}(t)\rangle$ is found to exhibit a cosinusoidal phase of 0 rad, i.e., $\langle R^{(1)}(t=0)\rangle$ is a maximum, and $\langle R\rangle$ is found to vary between 2.122 and 2.142 Å.

Supplementary Note 8: Retrieval of H–C–I bond angle for the CH_3I^+ I 4*d* core-excited state

The Huang-Rhys factor S_i for a particular vibrational mode i is related to the mean square displacement d_i by the expression

$$S_i = \frac{\omega_i}{2\hbar} d_i^2\,, \tag{5}$$

where ω_i is the vibrational frequency and d_i is the displacement corresponding to the difference between the normal coordinates of the excited (Q_i') and ground (Q_i) states:

$$d_i = Q_i' - Q_i = \sum_j L_{ij}\left(q_j' - q_j\right). \tag{6}$$

q_j' and q_j are the mass-weighted Cartesian coordinates of the excited and ground states respectively and L_{ij} are elements of the transformation matrix L, made up of the eigenvectors of the mass-weighted Hessian. We make an assumption that the configuration of the excited state lies directly along the normal mode of the ground state.

For the umbrella mode, we extract the transformation matrix from the output of the GAUSSIAN software package[2], which gives $\mathrm{I}_{\text{cart}} = \mathrm{ML}$, where M is a diagonal matrix with elements $M_{ii} = m_i^{-1/2}$.

	X (Å)	Y (Å)	Z (Å)
**C**	0.00	0.00	–0.11
**H**	0.00	0.19	0.54
**H**	–0.17	–0.10	0.54
**H**	0.17	–0.10	0.54
**I**	0.00	0.00	0.00

In order to calculate the angle, we first need to define the geometries q_j' and q_j. We use the description of the geometry where the C atom is placed at the origin, and the I atom is located directly above it along the positive *Z* direction.

	X (Å)	Y (Å)	Z (Å)
**C**	0	0	0
**H**	0	$d_{\mathrm{C-H}}\lvert\sin\theta\rvert$	$d_{\mathrm{C-H}}\lvert\cos\theta\rvert$
**H**	$-(\sqrt{3}/2)d_{\mathrm{C-H}}\lvert\sin\theta\rvert$	$-(1/2)d_{\mathrm{C-H}}\lvert\sin\theta\rvert$	$d_{\mathrm{C-H}}\lvert\cos\theta\rvert$
**H**	$(\sqrt{3}/2)d_{\mathrm{C-H}}\lvert\sin\theta\rvert$	$-(1/2)d_{\mathrm{C-H}}\lvert\sin\theta\rvert$	$d_{\mathrm{C-H}}\lvert\cos\theta\rvert$
**I**	0	0	$d_{\mathrm{C-I}}$

To calculate q_j', we evaluate all of the positions numerically using the experimental geometric parameters[13]:

Geometric parameter	Value
C—I bond length	2.126 Å
C—H bond length	1.091 Å
I–C–H bond angle	108.3°

Using the experimentally measured value of the vibrational frequency ($\omega = 1253$ cm^{-1}) and the measured value of the Huang-Rhys factor ($S = 8.3 \times 10^{-3}$), we can calculate the value of this displacement. We then calculate the change in the I–C–H bond angle by comparing the geometries of q_j' and q_j. Two solutions for the change in angle are possible: 106.5° and 110.0°. Based on the earlier argument that the experiment finds a larger angle, we

obtain a H–C–I bond angle of 110.0° for the I $4d_{5/2}^{-1}$ core-excited state of CH_3I^+. The estimated error of ±0.6° is obtained from the sum of the error of the experimentally determined H–C–I angle[13] of ±0.3° and the error in the Huang-Rhys factor.

Supplementary Methods: Details of the experimental setup

The experimental apparatus for femtosecond XUV absorption spectroscopy (Supplementary Figure 9a) is based on an amplified femtosecond laser system (Femtolasers GmbH) with an output center wavelength of 800 nm, pulse duration of 25 fs FWHM, and output pulse energy of 3 mJ at 1-kHz repetition rate. The laser output is spectrally broadened in a 1-m-long hollow-core fiber filled with 1.5 bar of helium gas before it is temporally recompressed by a set of chirped mirrors (Ultrafast Innovations GmbH). The visible-to-near-infrared (NIR) output from the chirped mirror compressor spans 553 – 962 nm at –10 dB (Supplementary Figure 9b), and has a carrier wavelength of 786 nm, a FWHM duration of 5.6 fs (Supplementary Figure 9c), and a pulse energy of 1.2 mJ.

The XUV probe pulse is produced by focusing 0.8-mJ of the few-cycle output into a 2-mm-long argon gas cell with a quasi-static pressure of 54 mbar to drive high-order harmonic generation (HHG). The residual visible-NIR laser beam is removed by a 0.2-μm-thick Al foil positioned after the HHG gas cell. The XUV beam is refocused by a toroidal mirror (ARW Optical) that is designed for 1:1 relay-imaging of the XUV source onto the sample target. After the sample target, the transmitted XUV beam passes through another 0.2-μm-thick Al foil before it enters a home-built XUV spectrometer.

The XUV spectrometer is equipped with a concave mirror (ARW Optical), a varied-line-spaced grating (Hitachi), and a thermoelectrically cooled X-ray CCD camera (Princeton Instruments). A typical XUV probe spectrum produced by HHG with argon is shown in Supplementary Figure 9d. The spectrometer is calibrated by observing the positions of the $^2P_{3/2} \rightarrow {}^2D_{5/2}$ and $^2P_{1/2} \rightarrow {}^2D_{3/2}$ transitions[16] of Xe^+, located at 55.39 and 56.08 eV,

respectively, the $4d^9(\ ^2D_{5/2})6p$, $4d^9(\ ^2D_{5/2})7p$, $4d^9(\ ^2D_{3/2})6p$, and $4d^9(\ ^2D_{3/2})7p$ resonances[17] of neutral Xe, located at 65.11, 66.37, 67.04, and 68.34 eV, respectively, and the aluminum L_3 edge[18] at 72.64 eV. The spectral resolution is determined from the fit of the Xe^+ $^2P_{3/2} \rightarrow\ ^2D_{5/2}$ transition to a Voigt profile (Supplementary Figure 10), where the Lorentzian linewidth is fixed as the natural linewidth of the transition (122 meV)[16] and the Gaussian width is determined by the spectrometer resolution. In this manner, the resolution is found to be 47 meV FWHM in the XUV photon energy range employed in the present study.

Pump pulses in the visible-NIR with 0.17-mJ energy and 5.6-fs FWHM duration are loosely focused by a 1-m focal length spherical mirror onto the sample target. The resultant peak intensity of 1.9×10^{14} W/cm^2 drives strong-field ionization of the sample. The sample target is a 3-mm path length quasi-static gas cell with 250-μm-diameter entrance and exit pinholes. The pump beam intersects the XUV probe beam at an angle of 1° at the sample target. For the present experiments, the gas cell is heated to 353 K and the CH_3I vapor pressure in the gas cell is 14 mbar. The CH_3I sample (Merck, >99% purity) was used as received. A variable time delay between the pump and probe pulses is introduced by means of a computer-controlled piezo-driven delay stage (Physik Instrumente GmbH) positioned in the path of the pump beam. At each time delay, the XUV spectra, $S(E,t)$, are accumulated over 0.5 s and referenced to the XUV spectrum collected at –500-fs time delay, $S(E,t = -500 \text{ fs})$, such that the differential absorption signal is given by $\Delta A(E,t) = -\log[S(E,t)/S(E,t = -500 \text{ fs})]$. The midpoint of the rise in the CH_3I^+ ($\tilde{X}^+\ ^2E_{3/2}$) parent ion signal at 47.0 eV, when the instantaneous ionization rate is the highest, is used to define time-zero to a precision of $\Delta t_0 = 1.2$ fs (Supplementary Figure 11). This timing jitter in turn contributes to the uncertainty in the initial phase of the wave packet-induced oscillation via $\Delta\phi = \omega\Delta t_0$, where ω is the oscillation frequency.

Supplementary References

1. Olney T. N., Cooper G., Brion C. E. Quantitative studies of the photoabsorption (4.5–488 eV) and photoionization (9–59.5 eV) of methyl iodide using dipole electron impact techniques. *Chem. Phys.* **232**, 211-237 (1998).
2. Frisch M. J., Trucks G. W., Schlegel H. B., Scuseria G. E., Robb M. A., Cheeseman J. R., *et al.* Gaussian 09, Revision B.01. Wallingford CT; 2009.
3. Chai J. D., Head-Gordon M. Systematic optimization of long-range corrected hybrid density functionals. *J. Chem. Phys.* **128**, 084106 (2008).
4. Weigend F., Ahlrichs R. Balanced basis sets of split valence, triple zeta valence and quadruple zeta valence quality for H to Rn: design and assessment of accuracy *Phys. Chem. Chem. Phys.* **7**, 3297-3305 (2005).
5. Saenz A. Enhanced ionization of molecular hydrogen in very strong fields. *Phys. Rev. A* **61**, 051402 (2000).
6. Posthumus J. H. The dynamics of small molecules in intense laser fields. *Rep. Prog. Phys.* **67**, 623-665 (2004).
7. Gedanken A., Rowe M. D. Magnetic circular dichroism spectra of the methyl halides. Resolution of the n $\rightarrow$ σ * continuum. *Chem. Phys. Lett.* **34**, 39-43 (1975).
8. Alekseyev A. B., Liebermann H.-P., Buenker R. J., Yurchenko S. N. An ab initio study of the CH_3I photodissociation. I. Potential energy surfaces. *J. Chem. Phys.* **126**, 234102 (2007).
9. Song Y., Qian X.-M., Lau K.-C., Ng C. Y., Liu J., Chen W. High-resolution energy-selected study of the reaction $CH_3X^+ \rightarrow CH_3^+ + X$: accurate thermochemistry for the CH_3X/CH_3X^+ (X=Br, I) system. *J. Chem. Phys.* **115**, 4095-4104 (2001).
10. Shimanouchi T. *Tables of molecular vibrational frequencies* consolidated Vol. I. (National Bureau of Standards, 1972)
11. Lee M., Bae Y. J., Kim M. S. *K* selection in one-photon mass-analyzed threshold ionization of CH_3I and CD_3I to the $\tilde{X}\,^2E_{3/2}$ state cations. *J. Chem. Phys.* **128**, 044310 (2008).
12. Matsuura H., Overend J. Equilibrium structure of methyl iodide. *J. Chem. Phys.* **56**, 5725-5727 (1972).
13. Bae Y. J., Kim M. S. Photodissociation spectroscopy of CD_3I^+ generated by mass-analyzed threshold ionization for structure determination. *ChemPhysChem* **9**, 1709-1714 (2008).
14. Duncan J. L., Ferguson A. M., Mathews S. Vibrational anharmonicity in CH_3I: a joint local and normal mode study. *J. Chem. Phys.* **91**, 783-790 (1989).
15. Ammosov M. V., Delone N. B., Krainov V. P. Tunnel ionization of complex atoms and of atomic ions in an alternating electromagnetic field. *Sov. Phys. JETP* **64**, 1191-1194 (1986).
16. Andersen P., Andersen T., Folkmann F., Ivanov V. K., Kjeldsen H., West J. B. Absolute cross sections for the photoionization of 4*d* electrons in Xe^+ and Xe^{2+} ions. *J. Phys. B* **34**, 2009-2019 (2001).
17. Ederer D. L., Manalis M. Photoabsorption of the 4*d* electrons in xenon. *J. Opt. Soc. Am.* **65**, 634-637 (1975).
18. Seely J., Kjornrattanawanich B. Measurement of extreme-ultraviolet attenuation edges of magnesium, tin, and indium filters. *Appl. Opt.* **42**, 6374-6381 (2003).
